# Analysis of polydisperse polymer adsorption on porous cellulose fibers

**DOI:** 10.1515/npprj-2023-0058

**Published:** 2024-04-29

**Authors:** Robert H. Pelton, Abdollah Karami, Jose Moran-Mirabal

**Affiliations:** Department of Chemical Engineering, McMaster University, 1280 Main Street West, L8S 4M1, Hamilton, ON, Canada; Department of Chemistry and Chemical Biology, McMaster University, 1280 Main Street West, L8S 4M1, Hamilton, ON, Canada

**Keywords:** adsorption isotherm, polymer adsorption, porous media, pulp fibers

## Abstract

The adsorption of cationic water-soluble polymers onto negatively charged porous wood pulp fibers is an essential aspect of papermaking. Adsorption data can be displayed as a direct plot of the amount adsorbed, Γ, versus the amount of polymer added or as an isotherm plot showing the amount adsorbed versus the residual unadsorbed polymer. In either data presentation, the analysis is more transparent if the units of each axis are the same (e.g., mg/g or meq/g), giving dimensionless slopes. Values for Γ_max_, Γ_I_, *f*_I_*,* and Γ_me_ can be extracted from many isotherms where: Γ_max_ is the maximum capacity of the fibers to adsorb polymer; Γ_I_ is the *y*-axis isotherm intercept and gives the maximum dose that can be fully adsorbed; *f*_I_ is the slope of the direct plot at Γ_I,_ and *f*_*I*_ is the mass fraction of the added polymer that can access interior (pore) surfaces; and, Γ_me_ is the saturated amount of polymer adsorbed on exterior surfaces. Additionally, the molecular weight distribution of the adsorbing polymer in conjunction with the adsorption isotherm can be used to estimate the molecular weight distributions of adsorbed polymer on interior and exterior fiber surfaces as functions of the polymer dose.

## 1 Introduction

Modern papermaking frequently employs cationic water-soluble polymers to improve the process and the final paper properties. Positively charged polymers spontaneously adsorb onto negatively charged wood pulp fibers in dilute aqueous suspension, efficiently modifying fiber properties before the fibers are filtered to form wet paper. Many studies of cationic polymer adsorption have been published, including excellent reviews, reflecting the importance of polymer adsorption in the papermaking process (Hubbe et al. 2009; van de Ven 2000; Wågberg 2000; Wågberg and Odberg 1989). Most adsorption results are presented as isotherms, which are plots of the amount of adsorbed polymer as a function of the concentration of unadsorbed polymer. However, isotherm interpretation can be complicated because many papermaking polymers are polydisperse in molecular weight and composition. Furthermore, wood pulp fibers are complex adsorption substrates because most of the cellulose/water interface is inside the porous fiber walls. This contribution proposes ways to extract information from studies of polydisperse polymer adsorption onto porous wood pulp fibers. We start with a brief overview of the relevant polymer adsorption literature.

Much of the significant theoretical and experimental polymer adsorption studies were done in the last quarter of the previous century when theoretical and experimental tools were quickly advancing, giving insights into the configuration of adsorbed polymer chains (Fleer et al. 1993; Pefferkorn 1999; van de Steeg et al. 1992). An important conclusion from the early studies is that the adsorption of high molecular weight polymers is irreversible. When a high molecular weight polymer chain has many segments in contact with a surface, desorption requires the improbable simultaneous detachment of all bound segments. Additionally, adsorbed high molecular polymers rarely form multilayers. The saturated adsorbed coverage, *λ*_max_ in adsorbed monolayers, usually range from 0.1 to 10 mg/m^2^. Kinetic effects such as diffusion rates, reconfiguration rates (Einarson et al. 1991), and the displacement of low molecular weight fractions by larger chains can influence *λ*_max_ (Cohen Stuart et al. 1984; Kawaguchi 1990; Schneider et al. 1996).

### 1.1 Polymers on wood pulp fibers

Wood pulp fibers are unsuitable substrates for many modern tools for probing polymer adsorption. For example, quartz crystal microbalances, surface plasmon resonance instruments, ellipsometry, or neutron reflectivity require substrates with smooth surfaces. Instead, most pulp fiber adsorption data involve the classic technique of adding polymer to a fiber suspension and filtration or centrifugation after a controlled mixing time. The unadsorbed polymer concentration in the fiber-free solution is measured by polyelectrolyte titration, UV absorbance, or possibly organic carbon and nitrogen contents. The results are given as the adsorbed polymer mass per dry fiber mass Γ. In some publications, Γ is expressed as milliequivalents of adsorbed charge divided by dry fiber mass, reflecting the use of charge titration to measure adsorbed or unadsorbed polymer. Multiplication of Γ (meq/g) by the equivalent weight of the adsorbing polymer yields a mass ratio.

To convert from Γ (mg/g) to *λ* (mg/m^2^), the appropriate specific surface area (SSA, m^2^/g) must be known – see Eq. (1). For porous wood pulp fibers, the specific surface area is a function of the size of the probe used to estimate SSA. Large probes only access the exterior fiber surfaces where SSA ∼1 m^2^/g, (Alince and Van de Ven 1997) a protein probe gave a value around 10 m^2^/g (Hong et al. 2007), and NMR using water molecules as a probe gave ∼100 m^2^/g (Larsson et al. 2013).

(1) Γ=λ·SSA

One approach to the dilemma of not knowing *λ* or SSA in Eq. (1) is to employ low porosity cellulose film substrates in polymer adsorption studies (Enarsson and Wågberg 2008). For example, many of the published adsorption isotherms involve PDADMAC (the full chemical names of polymers are given in the Abbreviations) because it has a high content of pH-independent quaternary ammonium groups, nominally a linear structure, and has commercial applications. However, commercial samples are branched and may associate in aqueous solution (Wandrey et al. 1999). A recent study with non-porous model cellulose films showed that PDADMAC gave *λ*_max_ values around 1 mg/m^2^ and was independent of molecular weight (Sampl et al. 2023). In 8 mM, NaCl *λ*_max_ only doubled over a large span of PDADMAC molecular weights. They also showed that PDADMAC samples from Sigma had bimodal particle size distributions.

Moving to fiber substrates, the following summary leans heavily on results from Tom Lindström and Lars Wågberg, who have worked for decades on polymer adsorption on pulp fibers. Cationic polymer adsorption on pulp fibers is driven by entropy gains from the release of counterions when positively charged polymer adsorbs near a carboxylic acid group. The saturation coverage of cationic polymer on negatively charged fibers estimates the fiber-accessible charge content (Wågberg et al. 1989).

Polymer adsorption onto fiber pores can be very slow – quoting (Horvath et al. 2008a,b,c), “High charge density polyelectrolytes were observed to diffuse on a time scale of months, whereas the diffusion of low charge density polyelectrolytes was measured on the order of hours.” The same paper argued that charge density and molecular size influence transport into the pores. Highly charged polymers are unlikely to enter pores without high salt concentrations (Horvath et al. 2008a,b,c). These results highlight the need to control adsorption times and mixing conditions and recognize that the maximum capacity of adsorb polymer, Γ_max_, is likely to be adsorption-time dependent.

The Wågberg group showed that the pre-adsorption of a high molecular weight fraction followed by a low MW polymer had little impact on the ability of the low MW polymer to enter the pores. Their first publication described two molecular weight fractions of PDADMAC (Wågberg and Hagglund 2001), whereas the second described cationic dextrans (Horvath et al. 2008a,b,c). These observations have two implications. First, it is possible to independently modify internal and exterior fiber properties by treatment with combinations of low and high molecular weight polymers. Second, when adsorbing a polydisperse polymer, the low molecular weight fractions can simultaneously adsorb on exterior and internal fiber surfaces. In summary, with polydisperse polymers typically used in papermaking, the adsorption isotherms reflect the distribution of polymer sizes and the fiber wall porosity.

The genesis of this contribution was our interest in modifying the adsorption capacity of fibers by grafting highly carboxylated polymers (Zhang et al. 2021). We realized the literature does not guide the adsorption data analysis from polydisperse polymers interacting with porous fibers. New approaches to isotherm analysis are proposed herein. The results section of this paper has four parts. First, alternative ways to display adsorption data are described. Second, a mass balance analysis gives a set of parameters that can be extracted from some isotherms, giving valuable insights for scientists and papermakers. Third, presented is a naive approach to estimating the maximum molecular weight of adsorbing polymers as functions of polymer dose. Fourth, mainly published experimental isotherms are used to demonstrate the proposed analysis.

## 2 Materials and methods

### 2.1 Materials

Polyamide-amine epichlorohydrin (PAE) resin (Kymene 777 LX, 12.50 % total solid) was provided by Solenis, US. The standard polyelectrolyte titrants poly(diallyldimethyl ammonium chloride), (PDADMAC, 0.001 M), and potassium polyvinyl sulfate (0.001 M) were purchased from BTG Americas Inc. (US). The pulp used in this study was dry lap ECF 90, northern bleached softwood kraft, provided by Canfor, Canada.

### 2.2 PAE adsorption

In the PAE adsorption experiments, dry wood pulp was dispersed in DI water to a consistency of 0.075 % using a disintegrator (20,000 revolutions). Then, the pulp slurry was filtered under vacuum on a Buchner funnel fitted with Whatman qualitative filter paper and resuspended in 1 mM NaCl solution to a consistency of 0.37 %.

The pH was adjusted to 7–8 and maintained in that range throughout the adsorption experiment. To start the adsorption experiment, PAE was added to the suspension and allowed to mix (500 rpm) for 15 min. After adsorption, the fibers were separated from the polymer solution using vacuum filtration, and the filtrate was analyzed by polyelectrolyte titration to determine the remaining unadsorbed PAE. The PAE content of the filtrate was determined by polyelectrolyte titration using a particle charge detector (PCD-03, Mütek). The adsorbed amount of PAE was calculated as the difference between the initial amount added and the amount remaining in the solution.

## 3 Results and discussion

### 3.1 The adsorption isotherm

**Figure 1:**
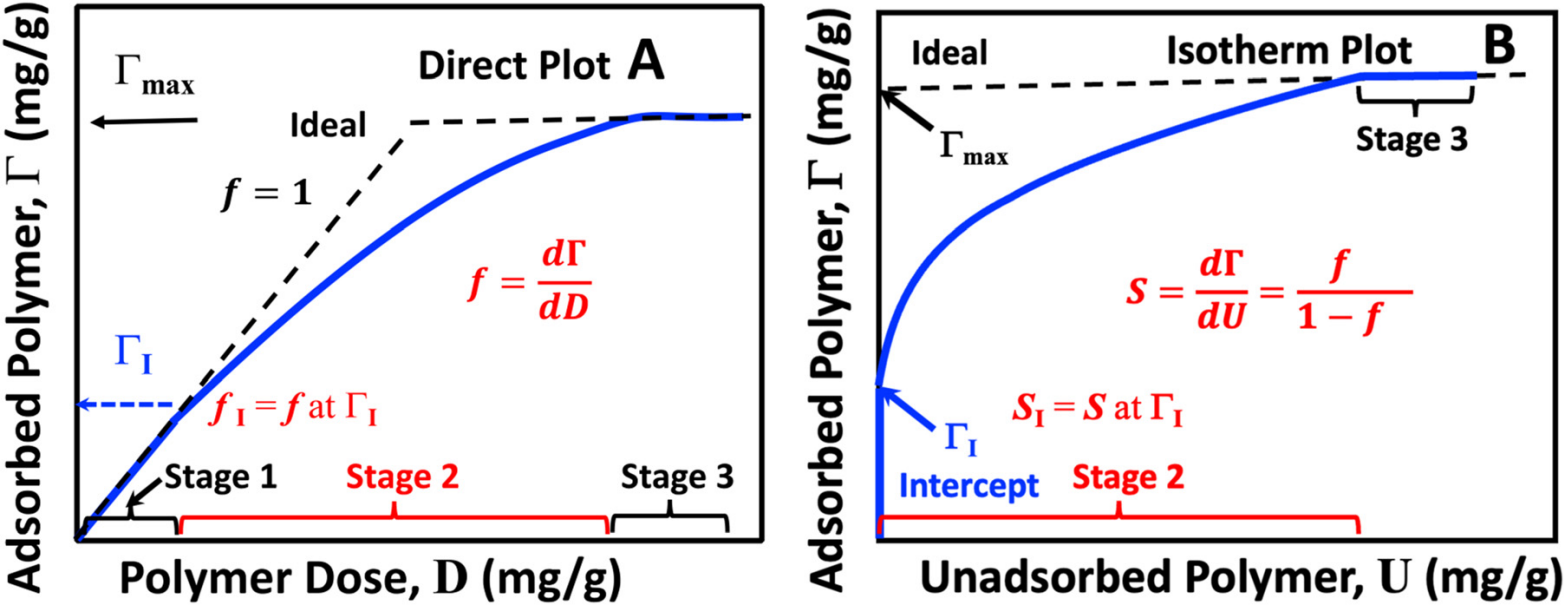
A schematic illustration of adsorption curves for irreversibly adsorbed water-soluble polymers on wood pulp fibers. The dashed lines give the corresponding ideal behaviors.

The most straightforward portrayal of polymer adsorption data is the “direct plot”, which is the quantity of adsorbed polymer Γ (mg of polymer/g of dry fiber) as a function of the dose of polymer, *D* (mg of added polymer/g dry fiber) – see Figure 1A. The adsorption curve can be divided into three stages. Stage 1 corresponds to low polymer dosages, where all the added polymer adsorbs until *D* = Γ_I_. In Stage 1, the slope of Figure 1A, *f* = 1. In Stage 2, some polymers adsorb, and some remain in the solution and 1 > *f* > 0. In Stage 3, the fibers are saturated, with Γ = Γ_max_ and *f* = 0. In theory, the ideal case indicated by the dashed lines in Figure 1A can be achieved by employing a uniform polymer with a narrow molecular weight distribution and ensuring the fiber/polymer contact times are sufficiently long to negate kinetic effects. There is no Stage 2 in the ideal case, and all added polymer is adsorbed until all accessible surfaces are saturated, after which there is no adsorption. The shape of the direct plot reflects the superposition of polymer property distribution with fiber pore accessibility distribution.

Although some published direct plots exist (Ampulski and Neal 1989; Huang et al. 2017; Obokata and Isogai 2007), the literature presents most polymer adsorption data as adsorption isotherms. Figure 1B shows the corresponding isotherm, a plot of Γ (mg/g) versus the unadsorbed polymer, *U* (mg/g). The isotherm plot has no new information; it is a different representation of the same data in Figure 1A. Many published adsorption isotherms, including ours (Zhang et al. 2001), expressed the unadsorbed polymer contents as a solution concentration such as *u* (mg/L). This is a carryover from traditional small molecule adsorption literature where solution concentrations were variables in reversible adsorption models. Since these models do not apply to irreversible polymer adsorption, we express adsorbed and unadsorbed polymer contents as the polymer mass per mass of dry fiber. Typically, fiber consistency, Con (g/L), is constant over the experiments used to create an adsorption isotherm. Therefore, the unadsorbed polymer concentration, *u* (mg/L), can be converted to *U* (mg/gm) by dividing by Con (g/L) – Eq. (2). Therefore, *U* reflects both the unadsorbed concentration and the fiber concentration during the adsorption experiments.

(2) $$U=\frac{u}{\text{Con}}$$

The direct and the isotherm plots in Figure 1 have dimensionless slopes. By contrast, the slope of an Γ (mg/g) versus *u* (mg/L) isotherm plot has the unintuitive units of L/g. Finally, the two plots in Figure 1 are linked by the mass balance *D* = Γ + *U*. Therefore, the Stage 2 slopes to the two forms of data presentations are linked by Eq. (3).

(3) $$\frac{d\Gamma }{dU}=S=\frac{f}{1-f}$$

*S*, the dimensionless isotherm slope varies from infinity, where all added polymer adsorbs in Stage 1 to zero in Stage 3. In contrast, the corresponding *f* value ranges from 1 in Stage 1 to zero in Stage 3. In the analysis of adsorption isotherms presented below, *S* values are converted to *f* values by Eq. (3) because the physical significance of *f* (1 to 0) is more transparent than *S* (∞ to 0). Finally, particularly deviant isotherms can have a negative slope in Stage 3. Negative *S* or *f* cases are not addressed herein.

Most isotherms are not ideal, and the presence of Stage 2 means some fraction of the added polymer chains are excluded from adsorbing on all the surfaces contributing to Γ_max_. An obvious explanation for exclusion is the presence of polymer molecules that are too large to enter small pores and, thus, are restricted to exterior surfaces. **Herein, we define “exterior surfaces” as those capable of adsorbing all fractions of the added polymer and “excluded polymer” as those polymer chains that can only access exterior surfaces**.

Polymer size is not the only exclusion mechanism. Compositional distributions in dosed polymer, insufficient time to allow polymer access to all surfaces, non-uniform fiber surface chemistry, crystalline versus amorphous surfaces, and the electrostatic repulsion between highly charged surfaces and polymers in low ionic strength solutions (Horvath et al. 2008a,b,c) are examples of other mechanisms that could prevent adsorption by some fraction of added polymer. To simplify the discussion, the following assumes that molecular size and the related molecular weight dictate penetration into fiber wall pores. However, most of the analysis below is independent of the exclusion mechanism.

Three and sometimes four parameters can be extracted from an adsorption isotherm. The first is Γ_I_, the *y*-axis intercept on the isotherm plot. Γ_I_ is the highest dose of added polymer (*D*_I_) that is completely adsorbed. Γ_I_ is also the upper limit estimate of the maximum quantity of adsorbed polymer on exterior surfaces.

The second parameter that can be extracted from most isotherms is *f*_I_, which is calculated from *S*_I_ by Eq. (3) where *S*_I_ is the limiting value of *S* when *U* approaches 0 (i.e., $S_{I}=\lim_{U\to 0}S$ ) in the isotherm plot. The direct plot in Figure 1A would yield a *f*_I_ value close to 1. However, we will see examples below where the isotherms appear to be a discontinuous function at Γ_I_, giving *f*_I_ values much less than 1. ***f***_**I**_ **is a valuable parameter because it corresponds to the maximum mass fraction of the added polymer that can access the interior (pore) surfaces**. We show examples of published isotherms with *f*_I_ values ranging from 0.12 for a high molecular weight polymer that mainly does not penetrate pores to *f*_I_ = 0.73 obtained with a low molecular weight adsorbing polymer.

The third isotherm parameter is Γ_max_, the maximum amount of adsorbed polymer. The fiber-specific surface area, accessible to the adsorbing polymer, can be estimated from Γ_max_ if the corresponding coverage, *λ*_max_, is known – see Eq. (1). Values for *λ*_max_ can be estimated by parallel adsorption experiments of smooth, non-porous cellulose films (Sampl et al. 2023). Alternatively, *λ*_max_ = 1 mg/m^2^ is a reasonable guess (Alince and Van de Ven 1997). We emphasize that Γ_max_ likely depends upon pH, ionic strength, mixing time, mixing intensity, and temperature. The conditions chosen for the adsorption experiments should be guided by the goals of the experiments and possibly the conditions of the commercial applications.

The fourth parameter **Γ**_**me**_ **is the saturated amount of polymer adsorbed on exterior surfaces**. Γ_me_ is an important property calculated from Γ_I_, Γ_max_, and *f*_I_*.* However, Γ_me_ values are model-dependent, as illustrated next.

### 3.2 Analyzing the adsorption isotherm

Experimental isotherms can yield accurate values for *f*_I_, Γ_I_, and Γ_max_. Presented now are parameters that can be calculated from these three experimental results. The most useful is Γ_me_ (mg/g), the saturation coverage of adsorbed polymer on exterior fiber surfaces. The determination of Γ_me_ from experimental data depends on mass transport details. We consider two cases, sequential adsorption and simultaneous adsorption. In sequential adsorption, the exterior surfaces are saturated before any pore adsorption. For sequential adsorption, Γ_me_ = Γ_I_, and the composition of the adsorbed polymer is roughly equal to that of the added polymer. At the other extreme, the simultaneous adsorption model assumes that lower molecular weight fractions can adsorb on interior surfaces in the same timescale as exterior surface adsorption. In this case, Γ_me_ < Γ_I_ because at the intercept Γ_I_, the adsorbed polymer is present in interior and exterior surfaces. The derivation in the Appendix is based on mass balances of adsorbed polymer at two points in the isotherm, Γ_I_ and at Γ_max_.

Table 1 gives expressions for various parameters derived from the experimentally accessible parameters Γ_I_, *f*_I_, and Γ_max_. Whereas we believe that simultaneous adsorption is closest to reality based on the literature (Horvath et al. 2008a,b,c; Wågberg and Hagglund 2001), the sequential adsorption expressions for Γ_me_ and *F*_Eme_ do not require knowledge of *Γ*_max_, which is an advantage when good values for *Γ*_max_ are unavailable. The application of the equations in Table 1 to experimental isotherms is presented later. Note Eqs. (13) and (14) only apply to constant-*f* isotherms.

**Table 1:** Isotherm parameters derived from Γ_I_, Γ_max_, and *f*_I_. Equations (6)–(18) are located in the Appendix.

Definition	Simultaneous adsorption	Sequential adsorption
The maximum amount of adsorbed polymer on exterior surfaces (mg/g)	$\Gamma_{\text{me}}=\frac{{\left(1-f_{I}\right)\cdot \Gamma }_{I}\Gamma_{\max}}{\Gamma_{\max\;-}f_{I}\Gamma_{I}}$ Eq. (9)	$\Gamma_{\text{me}}=\Gamma_{I}$ Eq. (16)
Mass fraction of excluded polymer on the saturated exterior fiber surface.	$F_{\text{Eme}}=1-\frac{{f_{I}\Gamma }_{I}}{\Gamma_{\max}}$ Eq. (12)	$F_{\text{Eme}}=1-f_{I}$ Eq. (17)
The smallest dose required to achieve Γ_max._	$D_{\max}=\frac{\Gamma_{\max}+\left(f_{I}-1\right)\cdot \Gamma_{I}}{f_{I}}$ Eq. (13)^a^	← Eq. (13)^a^
The maximum fraction of dosed polymer adsorbed at D_max._	$R_{\max}=\frac{{f_{I}\Gamma }_{\max}}{\Gamma_{\max}+\left(f_{I}-1\right)\cdot \Gamma_{I}}$ Eq. (14)^a^	← Eq. (14)^a^
The fraction of the total surface area contributing to Γ_max_ that is interior	$F_{\text{pore}}=\frac{\Gamma_{\max}-\Gamma_{I}}{\Gamma_{\max}-f_{I}\cdot \Gamma_{I}}$ Eq. (15)	$F_{\text{pore}}=\frac{\Gamma_{\max}-\Gamma_{I}}{\Gamma_{\max}}$ Eq. (18)

a Valid only for constant-*f* isotherms.

### 3.3 Linking polymer molecular weight distributions to adsorption isotherms

**Figure 2:**
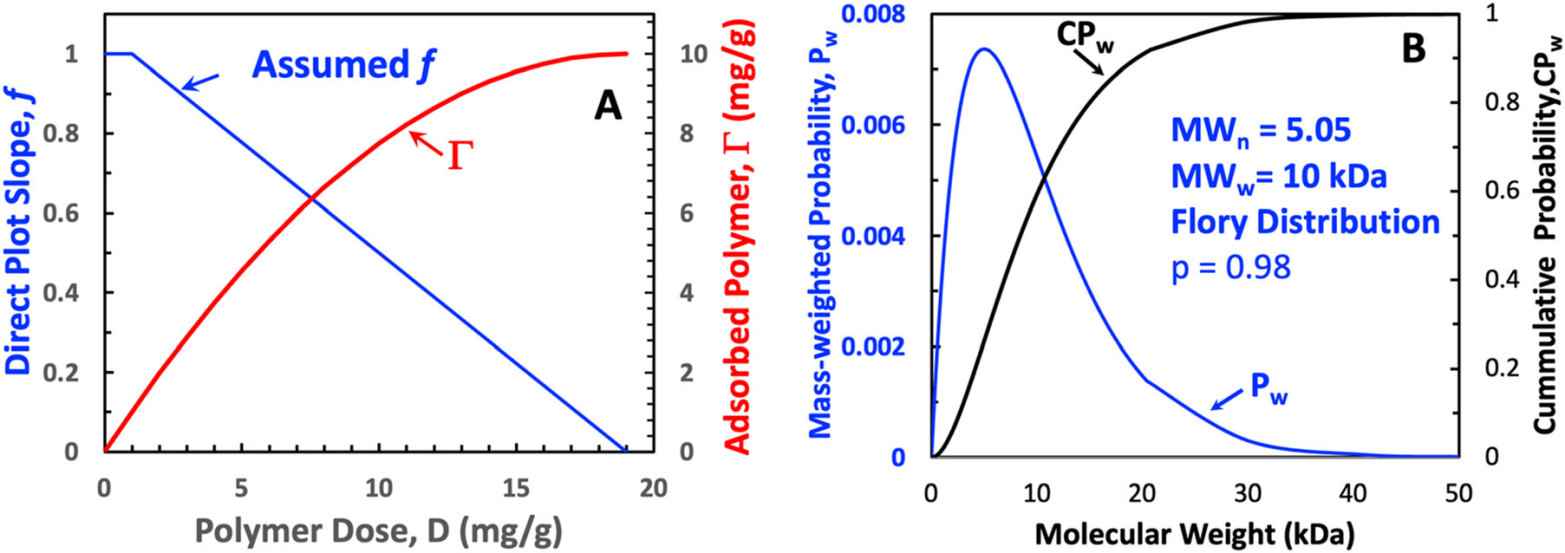
(A) Direct adsorption plot (Eq. (4)) for an assumed linear *f* function with fixed parameters Γ_I_ = 1 mg/g and Γ_max_ = 10 mg/g. (B) The simulated molecular weight distribution based on the Flory distribution (Eq. (5)) with a repeat unit MW of 100 Da.

This section presents a naive approach to estimating the maximum adsorbing molecular weight as it decreases over Stage 2 of an adsorption isotherm. The molecular weight distribution of the adsorbing polymer and the adsorption isotherms on porous fibers can be measured. However, for convenience, we employed the hypothetical curves in Figure 2. To generate adsorption direct plots or isotherms, we assumed *f* was a linear function of dose going from *f* = 1 and Γ_I_ = 1 mg/g to *f* = 0 at Γ_max_ = 10 mg/g. Figure 2A shows the assumed *f* function and the corresponding direct adsorption plot calculated with Eq. (4). Figure 2B shows the differential, *P*_w_, and the cumulative, CP_w_, mass-weighted molecular weight distributions for an ideal condensation polymer calculated from the Flory distribution given by Eq. (5) (Flory 1936). The parameters used in Eq. (5) are: *p* = 0.9802 is the assumed polymerization conversion; *n* is the number of monomer units in the polymer; and *z* = 100 Da is the molecular weight of the polymer repeat units.

(4) $$\Gamma \left(D\right)=\int_{0}^{D}fdD$$

(5) $${\text{MW}=z\cdot n\;\text{and}\;P}_{w}\left(n\right)=n\cdot p^{n-1}{\left(1-p\right)}^{2}\text{ and }{\text{CP}}_{w}\left(n\right)=\int_{0}^{n}P_{w}\left(n\right)dn$$

**Figure 3:**
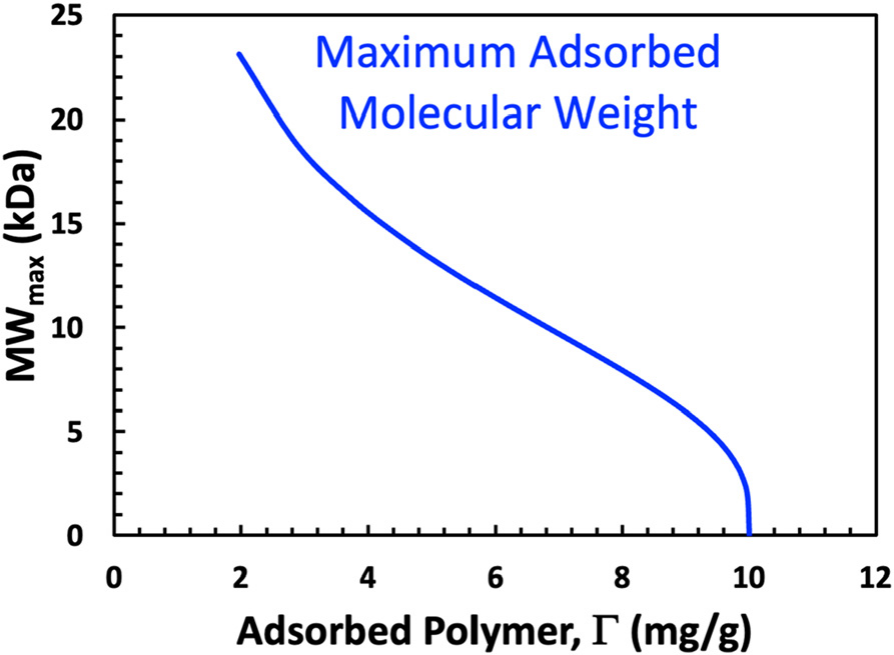
The maximum adsorbed polymer molecular weight, MW_max_, as a function of Γ. The analysis assumes that *f* = CP_w_(*n*) for every *f* value in Stage 2. The molecular weight distribution, CP_w_(*n*), and direct adsorption plots are shown in Figure 2.

Figure 2 shows two dimensionless functions that vary from 0 to 1, *f* the mass fraction of added polymer that adsorbs and CP_w_, the cumulative molecular weight distribution of the adsorbing polymer. Both *f* and CP_w_ reflect the size of the adsorbing polymer chains. One parameter influencing pore accessibility is the volume of solution occupied by a chain of the adsorbing polymer. The Mark-Houwink equation or a related function links chain volume to the chain molecular weight. For most papermaking polymers, the Mark-Houwink coefficients are not known. Therefore, we make the naive assumption that in Stage 2 of the adsorption process, *f* = CP_w_, linking the adsorption isotherms to molecular weight distribution. For example, we have a value for every value of *f* in Figure 2A, the corresponding molecular weight comes from Figure 2B, assuming *f* = CP_w_. The resulting plot of MW versus Γ is shown in Figure 3, where the molecular weight is labeled MW_max_. We propose that this curve represents the maximum adsorbing molecular weight for a given Γ value. Initially, when Γ is low or zero, the entire molecular weight distribution can adsorb. As the more accessible surfaces become occupied, only smaller polymer chains can adsorb. We acknowledge that assuming *f* = CP_w_ is speculative.

### 3.4 Analyzing experimental isotherms

**Figure 4:**
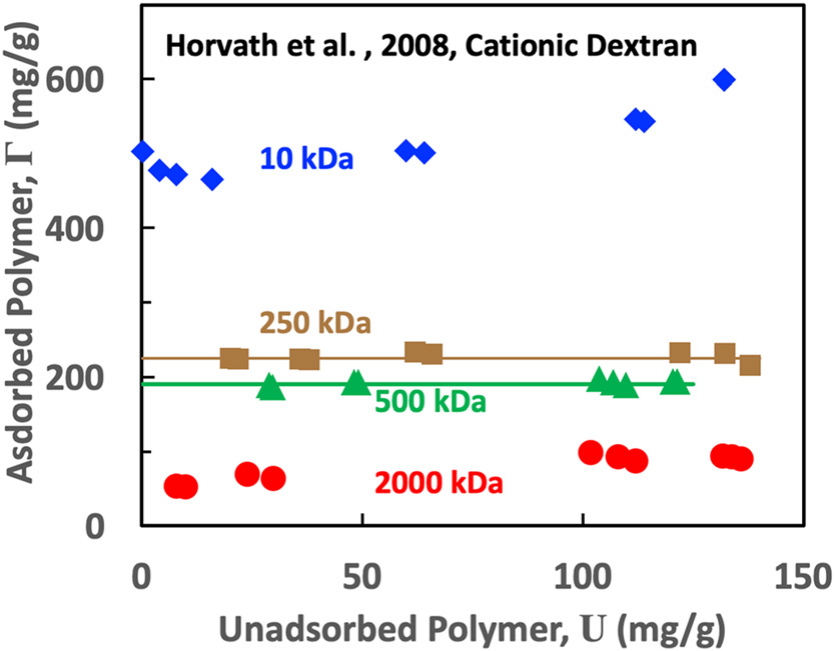
Cationic dextran adsorption isotherms on unbleached kraft pulps showing the influence of molecular weight on Γ_max_. These are examples of near-ideal horizontal isotherms. The data were extracted from Figure 3 in Horvath et al. (2008a,b,c).

The ideal adsorption isotherm consists of a vertical Stage 1, a horizontal Stage 3, and no Stage 2, and yields only a value for Γ_max_. Published horizontal isotherms for polymers on pulp fibers are rare. Figure 4 shows Horvath’s data converted via Eq. (2) to express the unadsorbed polymer in the same units as the *y*-axis. The two intermediate molecular weight curves in Figure 4 are horizontal lines. This result shows that higher molecular weight gives lower Γ_max_ values. Finally, these isotherms are unusual because quantities of adsorbed polymer are very high, whereas the unadsorbed polymer contents are low.

**Figure 5:**
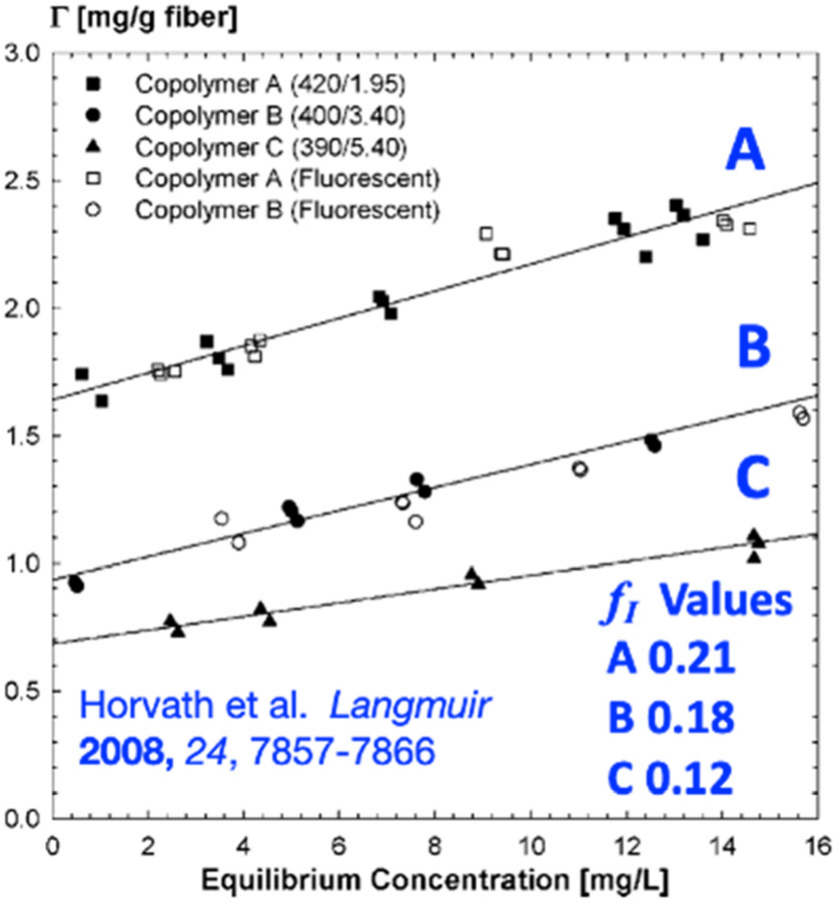
Examples of constant-*f* isotherms from the literature (Horvath et al. 2008a,b,c) showing the adsorption of three cationic polymers with similar molecular weight and varying charge densities with *C* > *B* > *A*. Used with permission of ACS.

Figure 5 shows three published isotherm plots for nearly equal molecular weight polymers but different charge densities (Horvath et al. 2008a,b,c). These are good examples of constant-S isotherm plots, which correspond to constant-*f* direct plots. Dimensionless slopes, *S*, of the isotherm plots were calculated by multiplying the slopes of the lines in Figure 5 by Con, the mass/volume concentration of pulp fibers used in the adsorption experiments. The resulting *S* = *S*_I_ slopes were converted to the corresponding *f*_I_ slopes by Eq. (3), and the *f*_I_ values are shown in Figure 5. Because there are no Γ_max_ results, we assume sequential adsorption giving Γ_me_ = Γ_I_ (Eq. (16)). Horvath used the three Γ_I_ values to illustrate the impact of charge density on Γ_I_. However, they did not analyze the slopes. All three *f*_I_ values are low (0.12–0.21), showing that most of the polymer could not enter interior surfaces. The *f*_I_ values did decrease by nearly a factor of two, going from the lowest to the highest charge density. We expect the highest charged density polymers to be the most expanded, which could explain the decreasing *f*_I_. The authors also reported confocal evidence of exterior fiber wall penetration and observed no penetration of the interior lumen wall. The *f*_I_ values give the fraction of added polymer adsorbing on interior surfaces, and the published confocal microscopy images show which interior surfaces are adsorbing polymer. Finally, note that this example emphasizes the role of charge density on the exclusion mechanism – the lower the charge density, the greater the fraction of added polymer that can contribute to Γ_max_.

**Figure 6:**
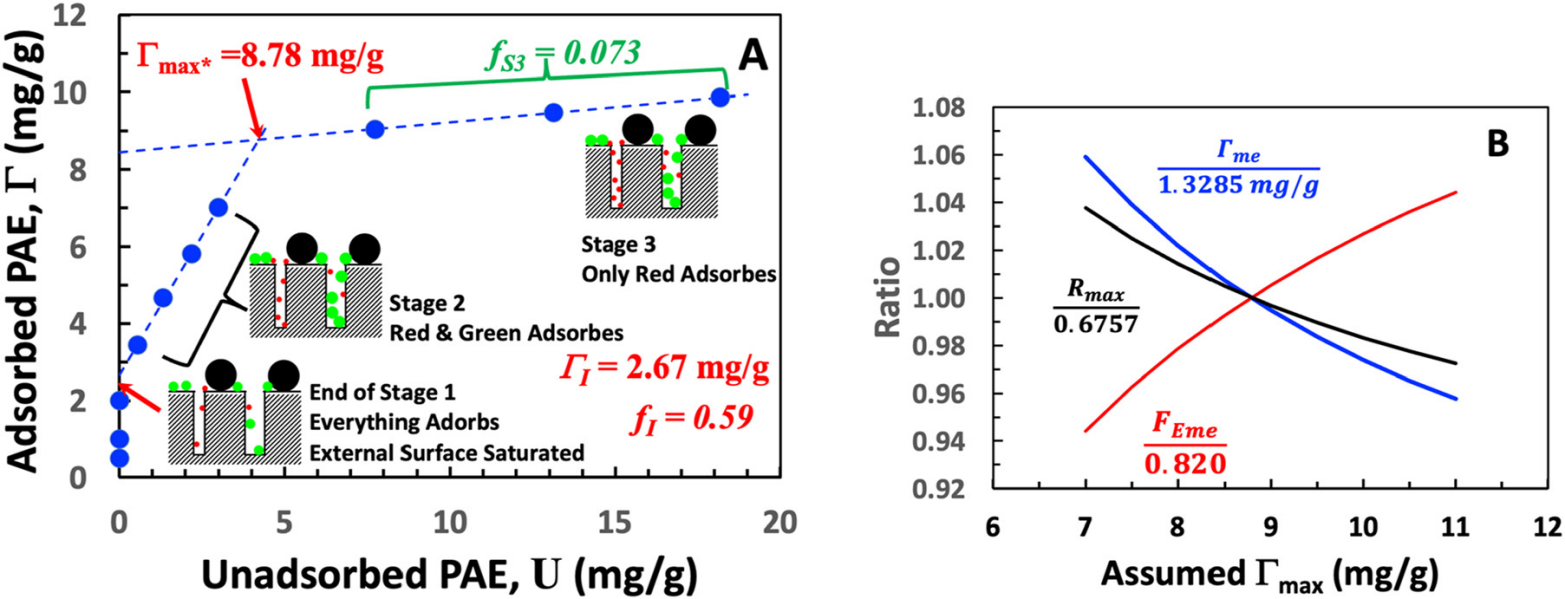
(A) The adsorption isotherm for PAE on softwood bleached kraft pulp. The PAE adsorption conditions were pH 7–8 and pulp concentration 3.7 g/L in 1 mM NaCl. (B) The influence of the assumed Γ_max_ value on *R*_max_, Γ_me_, and *F*_Eme_ (see Table 1) divided by their values at Γ_max_*.

Figure 6A shows our experimental isotherm for the adsorption of a commercial cationic wet-strength resin, PAE, on unbeaten softwood bleached kraft pulp. PAE is a modified condensation polymer with broad molecular weight distribution and crosslinks, giving a spherical conformation in solution (Obokata et al. 2005). Similar PAE direct plots have been reported in (Ampulski and Neal 1989). However, our analysis is new. The blue data in Figure 6A form a nearly ideal constant-*f* isotherm. The Stage 3 data had a low positive slope. The simplest physical model that gives this isotherm shape consists of fiber walls with small and large pores contacting a polymer that is a mixture of small, medium, and large polymer chains – see illustrations in Figure 6A. All three polymer sizes adsorb on the exterior surfaces. Only the medium and small polymers adsorb on the large fiber wall pores, leaving only the small polymers to adsorb on the small pore surfaces. The *f*_I_ value of 0.59 gives the fraction of dosed polymer capable of adsorbing on the large and small pores, and the *f*_*S*3_ value of 0.073 gives the mass fraction of polymer chains that can adsorb in the small pores.

Table 2 summarizes parameters that can be extracted from Figure 6A, where Γ_I_ is the *y*-axis intercept, *f*_I_ comes from the slope at the intercept, and *f*_*S*3_ comes from the slope of the Stage 3 line. The application of Table 1 models required Γ_max_. The potential Γ_max_ values range from the *y*-axis intercept value of 8.45 to 9.87 mg/g, corresponding to the largest dose. We applied the intermediate value, Γ_max_* = 8.78 mg/g, to the simultaneous adsorption model because it falls upon the linear Stage 2 line assumed by the model. The sensitivities of the calculated parameters to the value of Γ_max_ are shown in Figure 6B. To facilitate plotting the three parameters on the same graph, the values of *Γ*_me_, *F*_Eme,_ and R_max_ are divided by the value calculated from Γ_max_*. Over the assumed Γ_max_ range of 8–10 mg/g, Γ_me_, *F*_Eme,_ and *R*_max_ vary only by a couple of percent, showing that the calculated parameters are insensitive to the Γ_max_ values. Therefore, in cases where the isotherm’s high dose (Stage 3) regions are not horizontal, we employ Γ_max_* extracted from the data as shown in Figure 6A.

**Table 2:** Comparing predictions of sequential and simultaneous adsorption models for isotherm in Figure 6.

Experimental parameters (Figure 6A)	
*S*_I_ = *1.450*	
*f*_I_ = 0.592	
*f*_*S*3_ = 0.073	
Γ_I_ = 2.670 mg/g	
Γ_max_* = 8.78 mg/g	

**Simultaneous adsorption model**	**Sequential adsorption model**

$\Gamma_{\text{me}}=1.326\;\text{mg}/g$ Eq. (9)	Γ_me_ = Γ_I_ = 2.670 mg/g
$F_{\text{Eme}}=0.822$ Eq. (12)	$F_{\text{Eme}}=0.408$ Eq. (17)
$R_{\max}=0.675$ Eq. (14)	*R*_max_ = 0.675
$F_{\text{pore}}=0.851$ Eq. (15)	$F_{\text{pore}}=0.699$ Eq. (18)

What do the calculations in Table 2 tell us? Arguably Γ_me_, the maximum amount of wet-strength enhancing polymer on exterior fiber surfaces has the greatest impact on paper wet strength because wet paper usually fails at fiber/fiber joints. The simultaneous adsorption model, where adsorption occurs on all surfaces from the outset, predicts a Γ_me_ value that is half that predicted by the sequential model. Repeating an earlier statement – we believe simultaneous adsorption is closest to reality based on the literature (Horvath et al. 2008a,b,c; Wågberg and Hagglund 2001).

*F*_Eme_ is the fraction of Γ_me_ that is excluded polymer that only adsorbs on exterior surfaces. Thus, *F*_Eme_ is a measure of the exterior surface composition. The sequential value reflects the composition of the added polymer, whereas the simultaneous model predicts the mass fraction of the high molecular weight polymer is twice as high. Concentrating higher molecular weight components on the exterior surface should be desirable for wet-strength polymers.

*R*_max_ = 0.68 is the maximum fraction of added polymer that adsorbs at Γ_max*_. A papermaker would unlikely want 32 % unretained polymer, whereas with a dose equal to Γ_I_, potentially all the added polymer can be adsorbed.

Finally, *F*_pore_ = Γ_mp_/Γ_max_ is the fraction of Γ_max_ that is interior (pore) surfaces. The simultaneous value, 0.85, is greater than 0.67, predicted by sequential adsorption.

**Figure 7:**
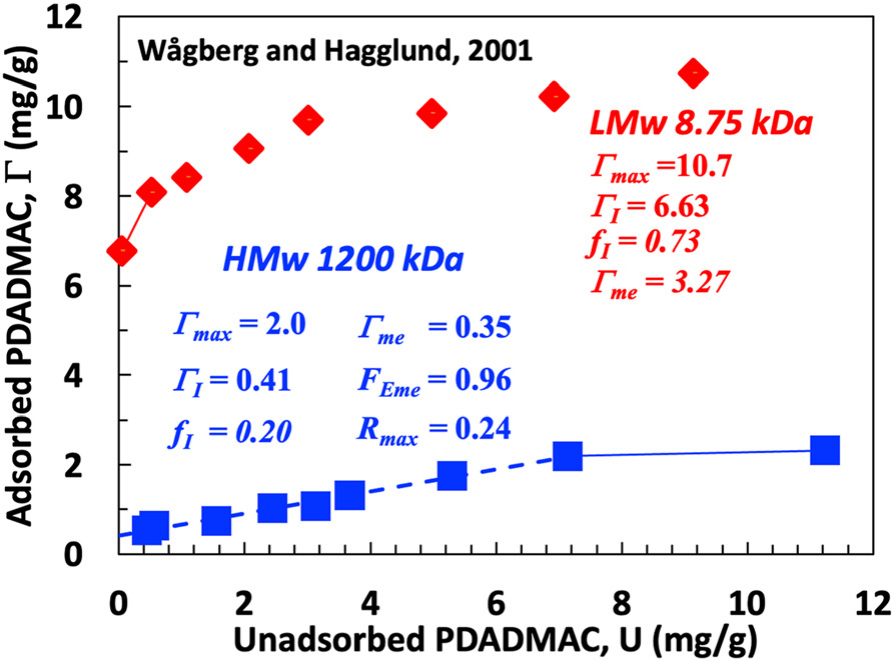
PDADMAC adsorption on a bleached softwood kraft pulp Figure 1 in Wågberg and Hagglund (2001). Calculated parameters are Γ_me_ from Eq. (9), *F*_eme_ from Eq. (12), and *R*_max_ from Eq. (14). All Γ values are in units (mg/g).

Further illustrating the application of the relationships in Table 1, Figure 7 shows two isotherm plots for PDADMAC adsorbed on bleached softwood kraft pulp, extracted from Figure 1 in Wågberg and Hagglund (2001). The *x*-axis units in the original publication were converted to mg/g by dividing by the experimental fiber concentration, Con = 5 g/L – see Eq. (2). Starting with the HM_w_ data, the high molecular weight PDADMAC isotherm is an excellent example of a constant-*f* isotherm. The left-hand column gives the values extracted from the isotherms, Γ_I_, *f*_I_ (via *S*_I_, Eq. (3)), and Γ_max_. The right-hand columns show the calculated parameters from the mass balance modeling (Table 1). The extrapolation of the two highest dose points is nearly horizontal, giving Γ_max_ = 1.98 mg/g. Only 24 % (the *R*_max_ value) of the minimum dose to achieve Γ_max_ was adsorbed. It is unlikely a papermaker would want such low retention. The maximum quantity of polymer on the exterior fiber surfaces was likely between Γ_me_ = 0.346 mg/g for simultaneous adsorption and Γ_I_ = 0.412 mg/g for sequential adsorption. The estimates are far lower than the extrapolated Γ_max_. The fraction of excluded, high molecular weight polymer on the exterior surfaces, *F*_Eme_ = 0.96, is slightly higher than that of excluded chains in the dosed polymer, 1 − *f*_I_ = 0.81.

The LMw isotherm in Figure 7 has a variable-*f* character. The intercept, *Γ*_I_ = 6.6 mg/g, is higher than 1.2 mg/g for the HM_w_ data. Γ_I_ and *f*_I_ values are based on the two left points with a line between them. Both isotherms in Figure 7 involve the same fiber type, and we now attempt to link the two plots in Figure 7.

Herein Γ_me_ is defined as the maximum amount of polymer adsorbed on exterior surfaces, defined as the surface area accessible to all fractions of the added polymer. Although this is an unambiguous definition, it is hampered by having Γ_me_ dependent upon both polymer and fiber properties. For example, the LMw PDADMAC adsorption gives the total exterior polymer as Γ_I_ = 3.27 mg/g, whereas HM_w_ gives 0.35 mg/g (Figure 7). Consequently, the largest fraction of LMw PDADMAC can access more surface area than the largest fraction of HM_w_ PDADMAC. It would be more beneficial to have a measure of the exterior surface that is only dependent on fiber properties. A pragmatic solution promoted by Wågberg et al. (1989) is to define exterior surfaces as those available to very high molecular weight polymers that are unlikely to penetrate fiber wall pores. Although the HM_w_ isotherm in Figure 7 is not ideal because some polymer chains enter the pores, Γ_me_ = 0.35 mg/g is a reasonable estimate for saturated polymer content on the exterior surfaces.

Researchers measure high *M*_w_ PDADMAC adsorption on wood pulp fibers is to estimate surface charge density on the exterior surfaces of fibers (Wågberg et al. 1989). If we assume the specific surface area (SSA) of the external fiber surfaces is 1 m^2^/g, (Alince and Van de Ven 1997) and that Γ_me_ = 0.35 mg/g corresponds to a stoichiometric charge balance, the pulp surface charge density from Wågberg’s HMw data is 2.1 μeq/g. If we further assume that the surface charge density on interior and exterior surfaces are the same, the SSA accessible to the LMw PDADMAC is 9.5 m^2^/g, which seems reasonable.

To summarize, our ability to exact information from a polymer adsorption isotherm depends upon the form of the isotherm and the density of reliable data. We have identified the following cases:

***The ideal isotherm with no Stage 2***. The ideal isotherm has no Stage 2, and Γ_I_ = Γ_max_. The horizontal isotherm yields only one quantity, Γ_max_. Figure 4 may show examples, although there is little data near the *y*-axis. Ideal isotherms involving fiber substrates appear to be rare.

***A constant-f isotherm over most of Stage 2 plus a convincing value for Γ***_**max**_. The HMw data in Figure 7 and the PAE isotherm in Figure 6 are examples. In cases where the Stage 3 results have a slight positive slope, an alternative value for Γ_max_ is Γ_max*,_ which is the intercept of the constant slope line with the Stage 3 line – see Figure 6A.

***A constant-f isotherm with no Γ***_**max**_. There are many examples of this in the literature, including Figure 5. These isotherms yield good values for Γ_I_ and *f*_I_, particularly if data is on and near the *y*-axis.

***A variable-f isotherm with f***_***I***_ ***significantly less than 1***. In this case, the slope of the direct plot, *f*, is a discontinuous function of dose at *D*_I_ = Γ_I_. The LMw data in Figure 7 may be an example; however, the data are sparse near the intercept. This case yields a measurable Γ_I_ and usually a Γ_max_. Γ_I_ give the maximum amount of polymer that can be completely adsorbed and is an upper estimate of the amount of polymer on the exterior surface Γ_me_. *f*_I_ is the maximum mass fraction of the added polymer that can access interior (pore) surfaces.

***A variable-f isotherm with f***_**I**_ ***values very close to 1***. This case occurs when a low mass fraction of exterior polymer is present, lowering Γ_I_ from Γ_max_. In this case, the adsorption isotherm has an asymptotic approach to the *y*-axis. The schematic plots in Figure 1 are variable-*f* isotherms with *f*_I_ values close to one. However, we have not found a good example of this case in the literature.

Finally, as shown in a previous section, if we know the cumulative molecular weight distribution of the adsorbing polymer and *f* as a function of dose, we can estimate the evolution of the MW_max_, the maximum adsorbed molecular weight as a function of the amount of adsorbed polymer – see Figure 3. This analysis can be applied to any shape of isotherm if the polymer molecular weight distribution is available.

## 4 Concluding remarks

Many of papermaking’s most important commercial polymers have broad molecular weight and composition distributions. This contribution guides the interpretation of adsorption isotherms involving polydisperse polymers and porous wood pulp fibers. We propose that the analysis applies to the adsorption onto other porous substrates. However, most of the analysis also applies to other exclusion mechanisms whereby a fraction of the added polymer cannot adsorb on all accessible surfaces. Examples include compositional distributions in dosed polymer, insufficient time to allow polymer access to all surfaces, non-uniform fiber surface chemistry, crystalline versus amorphous surfaces, and the electrostatic repulsion between highly charged surfaces and polymers in low ionic strength solutions.

Adsorption results can be expressed as a “direct plot” giving the amount of adsorbed polymer on fibers Γ (mg/g) versus the dosage *D* (mg/g). The slope of the direct plot, *f*, is dimensionless and usually varies from 1 at low dosage to 0 when the fibers are saturated – see Figure 1A. The corresponding “isotherm plot” shows Γ verses unadsorbed polymer, *U* (mg/g) with a dimensionless slope *S* – Figure 1B. The slopes of the two types of adsorption plots are related by *S* = *f*/(1 − *f*). Many published isotherms express the quantity of unadsorbed polymer as a concentration (e.g., mg/L); we believe this practice needlessly obscures the analysis.

Adsorption isotherms from broadly dispersed polymers are rich with details. The information that can be extracted from adsorption isotherms includes:

(1) Γ_I_ is the amount of adsorbed polymer corresponding to the *y*-axis intercept and is the fibers’ maximum capacity to adsorb all added polymer molecules. Γ_I_ is also the upper estimate of Γ_me_, the saturation coverage of adsorbed polymer exterior fiber surfaces.
(2) *f*_I_ is the maximum mass fraction of the added polymer that can access interior (pore) surfaces. When *f*_I_ is low, most added polymer chains do not penetrate the fiber wall pores. *f*_I_ can be calculated (Eq. (3)) from *S*_I_, the slope of the isotherm plot when the unadsorbed polymer content approaches the *y*-axis intercept. We found no publications calculating or emphasizing the significance of *f*_I_.
(3) Γ_max_ is the maximum content of the adsorbed polymer and is usually associated with high concentrations of unadsorbed polymer.
(4) Γ_me_ is the saturation coverage of the exterior fiber surfaces. Γ_me_ can be much less than Γ_max_ and somewhat less than Γ_I_ for polydisperse adsorbing polymers. Γ_me_ depends upon topochemical pathways. For sequential adsorption, where all added polymer saturates the exterior surfaces before any polymer enters the pores, Γ_me_ = Γ_I_ giving an upper limit estimate of the amount of adsorbed polymer on saturated exterior fiber surfaces. Alternatively, in the case of simultaneous adsorption, where adsorption occurs on all surfaces at the same time, some of the adsorbed polymer at Γ_I_ will be on interior pore surfaces, and Γ_me_ < Γ_I_. Γ_me_ can be calculated from Eq. (9) using parameters extracted from the isotherm.
(5) An estimate of the maximum molecular weight of the adsorbing polymer at any point in the isotherm comes from assuming that *f*, the slope of the direct plot, is equal to the corresponding cumulative mass-weighted molecular weight probability of the adsorbing polymer, CP_w_.

Finally, for those planning to perform adsorption measurements involving porous fibers – collecting much data near and on the *y*-axis of the isotherm plots will yield more accurate Γ_I_ and *f*_I_ values. Collecting high-dosage data yields Γ_max_ values, which can be applied to calculate the parameters in Table 1.

Future contributions will describe the applications of this isotherm analysis to PAE adsorption onto fibers whose surfaces have been modified by grafted anionic polymers.

## Abbreviations

### The adsorption isotherm

Con: Pulp fiber consistency (mg dry fiber per liter of pulp suspension).
*D*
: The polymer dose (mg dry polymer/g dry pulp fiber).
*D*_I_
: The polymer dose at the intercept. *D*_I_ = Γ_I_.
*D*_max_
: The minimum dose giving complete coverage, Γ_max_ (mg/g).
*F*_Eme_
: Mass fraction of excluded polymer on the saturated exterior fiber surface.
*f*
: The slope of the direct adsorption plots.
*f*_I_
: The slope of direct adsorption plots at the beginning of Stage 2. *f*_I_ is the maximum mass fraction of the added polymer that can access interior (pore) surfaces contributing to Γ_max_.
*f*_*S*3_
: The slope of direct adsorption plots at the beginning of Stage 3.
*F*_pore_
: Γ_mp_/Γ_max_ is the fraction of Γ_max_ that is interior (pore) surfaces.
MW_max_: The maximum molecular weight of the adsorbing polymer.
*R*_max_
: The maximum fraction of dosed polymer adsorbed at *D*_max._
SSA: Specific surface area (m^2^/g).
*S*
: The slope of isotherm plots.
*S*_I_
: The slope of the isotherm at the intercept with the *y*-axis.
*U*
: Unadsorbed polymer concentration expressed as (mg/g of dry fiber).
*u*
: Unadsorbed polymer concentration expressed as (mg/L).
Γ: The adsorbed polymer content (mg of polymer/g of dry fiber).
Γ_I_: The *y*-axis intercept of the adsorption isotherm corresponds to the maximum polymer dose where all the added polymer is adsorbed (mg/g).
Γ_max_: The maximum adsorbed polymer content (mg/g).
Γ_max*_: The interpolated Γ_max_ when the high dose data are not horizontal – see Figure 6 (mg/g).
Γ_me_: The maximum amount of adsorbed polymer on exterior surfaces (mg/g).
Γ_mp_: The maximum quantity of adsorbed polymer in the pore surfaces (mg/g).
Γ_pi_: The amount of polymer adsorbed on exterior surfaces when the *D* = Γ_I_ (mg/g).
*λ*
: The adsorbed coverage of polymer on cellulose (mg/m^2^).
*λ*_max_
: The saturated adsorbed coverage of adsorbed polymer (mg/m^2^).

### The Flory distribution

CP_w_: The cumulative mass-weighted molecular weight probability.
*n*_max_
: The number of repeat units in MW_max_.
*P*_w_
: The mass-weighted molecular weight probability.
*z*
: The molecular weight of a polymer repeat unit (100 Da).

### Polymers

PAE: Polyamide-amine epichlorohydrin.
PDADMAC: Poly(diallyldimethyl ammonium chloride).

## Figures and Tables

**Figure A1:**
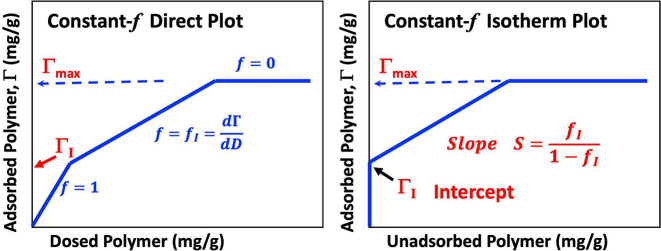
Direct and isotherm plots of a constant-*f* isotherm.

## Appendix

### Mass balances analysis of adsorption isotherms

This section develops expressions, giving Γ_me_ as a function of experimental Γ_I_, Γ_max_, and *f*_I_. Γ_max_ is the sum of two Γ_me_ and Γ_mp_, Eq. (6), where Γ_mp_ is the maximum amount of polymer on interior surfaces (subscript *p* for pores), and Γ_me_ is the maximum amount of adsorbed polymer on exterior surfaces. For most cases, Γ_mp_ > Γ_me_ reflecting the high specific surface area of fiber wall pores. When all accessible surfaces are saturated, the following mass balance applies (Figure A1).

(6)
$$\Gamma_{\max}=\Gamma_{\text{me}}+\Gamma_{\text{mp}}$$

At Γ_I_, we can write another mass balance (Eq. (7)), where Γ_pi_ is the amount of polymer adsorbed in pores when the polymer dose is *D* = Γ_I_. Note that for porous fibers where most of the surface area is in the pores, Γ_pi_ << Γ_mp_ because the pore surfaces are not saturated with adsorbed polymer when the dose is Γ_I_.

(7)
$$\Gamma_{I}=\Gamma_{\text{me}}+\Gamma_{\text{pi}}$$

Topochemical pathways determine the value of Γ_pi._ At one extreme, sequential adsorption, the exterior surfaces are saturated before the polymer enters pores, giving Γ_pi_ = 0 and Γ_I_ = Γ_me_, and the composition of the exterior adsorbed polymer exactly matches the added polymer. At the other extreme, simultaneous adsorption occurs on exterior and pore surfaces. When the exterior surface is saturated with adsorbed polymer at Γ_I_, there is also adsorbed polymer in fiber pore surfaces exterior surfaces. Therefore Γ_pi_ > 0 and Γ_me_ < Γ_I_.

### Analysis of simultaneous adsorption

At intercept Γ_I_, two types of adsorbed polymer are present: excluded polymer that can only adsorb on exterior surfaces and accessible polymer that can adsorb on all surfaces, contributing to Γ_max_. At Γ_I,_ the quantity of adsorbed accessible polymer is the product Γ_I_ *f*_I_. We assume that the accessible polymer will uniformly distribute between pores and the exterior surface in proportion to the relative areas of the two surface areas. Γ_mp_/Γ_max_ is the fraction of fiber surface area present as pore surfaces. Therefore, the quantity of accessible polymer entering the pores at the intercept is given by Eq. (8).

(8)
$$\Gamma_{\text{pi}}=\Gamma_{I}\cdot f_{I}\cdot \frac{\Gamma_{\text{mp}}}{\Gamma_{\max}}=\Gamma_{I}\cdot f_{I}\cdot \frac{\Gamma_{\max\;-}\Gamma_{\text{me}}}{\Gamma_{\max}}$$

Substituting Eq. (8) into Eq. (7) and rearranging gives Eq. (9) an expression for Γ_me_ in terms of Γ_max_, Γ_I_, and *f*_I_.

(9)
$$\Gamma_{\text{me}}=\frac{{\left(1-f_{I}\right)\cdot \Gamma }_{I}\Gamma_{\max}}{\Gamma_{\max\;-}f_{I}\Gamma_{I}}$$

The total adsorbed accessible polymer (i.e. low molecular weight) adsorbed when *D* = Γ_I_ is *f*_I_ Γ_I_. The corresponding adsorbed low molecular polymer on the exterior surface is given by the following based on the assumption that polymer partitions uniformly over interior and exterior surfaces.

(10)
$$f_{I}\Gamma_{I}\frac{\Gamma_{\text{me}}}{\Gamma_{\max}}$$

The fraction of low MW polymer on the exterior surface is:

(11)
$$\frac{f_{I}\Gamma_{I}\frac{\Gamma_{\text{me}}}{\Gamma_{\max}}}{\Gamma_{\text{me}}}=\frac{{f_{I}\Gamma }_{I}}{\Gamma_{\max}}$$

The corresponding fraction of excluded (high molecular weight) polymer on the saturated exterior surfaces is given by Eq. (12).

(12)
$$F_{\text{Eme}}=1-\frac{{f_{I}\Gamma }_{I}}{\Gamma_{\max}}$$

If the isotherm is the constant-*f* type, the minimum dose required to saturate all surfaces is

(13)
$$D_{\max}=\Gamma_{\max}+\left(1-f_{I}\right)\left(D_{\max}-\Gamma_{I}\right)=\frac{\Gamma_{\max}+\left(f_{I}-1\right)\cdot \Gamma_{I}}{f_{I}}$$

*R*_max_ is defined as the mass fraction of dosed polymer that is adsorbed at *D*_max_ and is given by Eq. (14).

(14)
$$R_{\max}=\frac{\Gamma_{\max}}{D_{\max}}=\frac{{f_{I}\Gamma }_{\max}}{\Gamma_{\max}+\left(f_{I}-1\right)\cdot \Gamma_{I}}$$

Finally, *F*_pore_ is the fraction of Γ_max_ that is accessible pores.

(15)
$$F_{\text{pore}}=\frac{\Gamma_{\text{mp}}}{\Gamma_{\max}}=\frac{\Gamma_{\max}-\Gamma_{\text{me}}}{\Gamma_{\max}}=\frac{\Gamma_{\max}-\Gamma_{I}}{\Gamma_{\max}-f_{I}\cdot \Gamma_{I}}$$

Summarizing the above set of equations gives the parameters in Table 1 of the paper, based on three experimental quantities, Γ_I_, Γ_max_, and *f*_I_, assuming simultaneous adsorption and a constant-*f* isotherm.

### Sequential adsorption

The analysis is simpler when the added polymer adsorbs onto and saturates the exterior surfaces before entering any pores. For the exterior fiber properties Γ_pi_ = 0, therefore:

(16)
$$\Gamma_{\text{me}}=\Gamma_{I}$$

The fraction of excluded high molecular weight polymer is equal to that of the dosed polymer

(17)
$$F_{\text{Eme}}=1-f_{I}$$

The following equation gives the fraction of the total accessible surface area that is internal.

(18)
$$F_{\text{pore}}=\frac{\Gamma_{\text{mp}}}{\Gamma_{\max}}=\frac{\Gamma_{\max}-\Gamma_{I}}{\Gamma_{\max}}$$
